# Automated detection of mouse scratching behaviour using convolutional recurrent neural network

**DOI:** 10.1038/s41598-020-79965-w

**Published:** 2021-01-12

**Authors:** Koji Kobayashi, Seiji Matsushita, Naoyuki Shimizu, Sakura Masuko, Masahito Yamamoto, Takahisa Murata

**Affiliations:** 1 Department of Animal Radiology, Graduate School of Agricultural and Life Sciences, The University of Tokyo, 1-1-1, Yayoi, Bunkyo-ku, Tokyo, 113-8657 Japan; 2 Autonomous Systems Engineering Laboratory, Graduate School of Information Science and Technology, Hokkaido University, Sapporo, Japan

**Keywords:** Computational biology and bioinformatics, Immunology, Physiology, Signs and symptoms

## Abstract

Scratching is one of the most important behaviours in experimental animals because it can reflect itching and/or psychological stress. Here, we aimed to establish a novel method to detect scratching using deep neural network. Scratching was elicited by injecting a chemical pruritogen lysophosphatidic acid to the back of a mouse, and behaviour was recorded using a standard handy camera. Images showing differences between two consecutive frames in each video were generated, and each frame was manually labelled as showing scratching behaviour or not. Next, a convolutional recurrent neural network (CRNN), composed of sequential convolution, recurrent, and fully connected blocks, was constructed. The CRNN was trained using the manually labelled images and then evaluated for accuracy using a first-look dataset. Sensitivity and positive predictive rates reached 81.6% and 87.9%, respectively. The predicted number and durations of scratching events correlated with those of the human observation. The trained CRNN could also successfully detect scratching in the hapten-induced atopic dermatitis mouse model (sensitivity, 94.8%; positive predictive rate, 82.1%). In conclusion, we established a novel scratching detection method using CRNN and showed that it can be used to study disease models.

## Introduction

Since the behaviour of experimental animals reflects their mental, physical, and cognitive status, it is often assessed in various fields of research. Experimental animals exhibit many behaviours, and researchers focus on specific phenotypes according to their interests. For example, in experimental rodents, the basic condition is assessed by observing spontaneous locomotor activity^1^, curiosity is assessed by observing rearing behaviours, and abdominal pain is assessed according to writhing behaviour^2,3^.

Scratching is one of the most important behavioural traits. Physiological disorders, as well as mental stress induce an itching sensation that manifests as scratching behaviour in animals. Several methods exist for measuring scratching, such as visual observation, acoustic detection, and induction current detection generated by the scratching motion^4–6^. These methods are accurate and are widely used in research. However, visual observation is time-consuming and labour-intensive while the acoustic detection and induction current detection methods require specialized equipment and complex analytical software. Therefore, a novel automated method that can detect scratching using simple equipment is required.

The recent development of deep neural network (NN) technologies has had a remarkable impact on animal research. Convolutional neural network (CNN), which effectively extracts feature maps from images, can deliver outstanding performance in image classification tasks^7^. Several studies have shown that CNN-based algorithms are able to predict an animal pose from images with very high accuracy^8–10^. Currently, several algorithms for pose estimation, such as LEAP and DeepLabCut, are available. Recurrent neural network (RNN), which can process time-series data, has also attracted attention. Since RNN can accept temporal and sequential inputs, it is used in machine translation and speech recognition^11,12^. Based on these findings, we hypothesized that a combination of CNN and RNN could be used to analyse movies, which are essentially a time-series integration of images.

Here, we show that a convolutional recurrent neural network (CRNN) trained with a total of about three hours of video can successfully detect scratching in mice in first-look videos with a high degree of accuracy. We also show that our trained CRNN can be used to assess pathological murine models.

## Results

### Image pre-processing and integration

**Figure 1:**
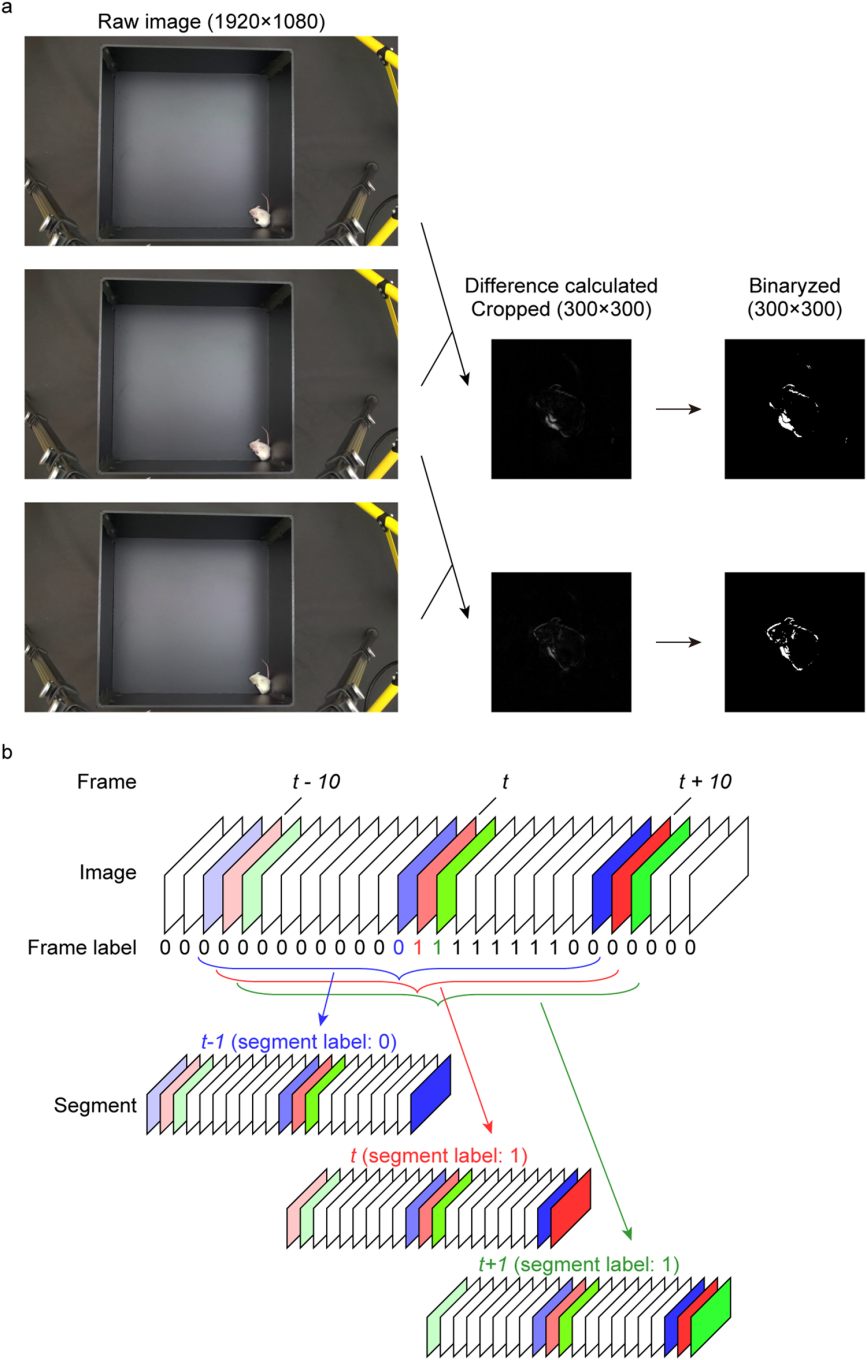
Image pre-processing and integration. (**a**) Video file was divided into the images of each frame. The absolute difference of each pixel between two continuous frames was calculated and cropped in a square shape (300 × 300 pixel) around the geometric centre of mouse. Images were then grey-scaled and binarized. (**b**) For segment at time *t*, the pre-processed images from *t − *10 to *t* + 10 was collected and labelled with the value of frame label at time *t*. The figure shows segment at *t − 1* (including images from *t *− 11 to *t* + 9, labelled as “0”), segment at *t* (including images from *t − *10 to *t* + 10, labelled as “1”), and segment at *t* + 1 (including images from *t − *9 to *t* + 11, labelled as “1”).

To induce typical scratching in mice, we intradermally administered a pruritogen, lysophosphatidic acid (LPA, 200 nmole/site/25 μL, 2 site/mouse) in the back of BALB/c mice (n = 9). We recorded their behaviour in a black arena using a handy camera and obtained 30 video files of about nine minutes each. The recorded videos contain background information unrelated to mouse movement, which increases the file size and also prevents efficient NN training. To eliminate the background and reduce the file size, we obtained differential images between two consecutive frames. Then, we cropped the images around the geometric centre of the mouse calculated as previously reported^13^ and binarized it (Fig. 1a). We grouped an image at time *t* and images ten frames before and after time *t* (Fig. 1b). The set of combined images was defined as the “segment” at time *t*.

### Segment labelling

We carefully observed the videos and categorized all frames into “scratching” and “not scratching” classes. All scratching frames were labelled “1” and the others were labelled “0”. Next, we annotated all segments by labelling a segment at time *t* as the value of the frame label at time *t* (Fig. 1b). Thus, we classified all segments into two classes: 1, scratching, and 0, not scratching. We then aimed to solve this binary classification problem using a NN. We randomly split 30 video files into 20 and 10 files, which were then used as the training and test datasets, respectively (Supplementary Table S1).

### CRNN training

**Figure 2:**
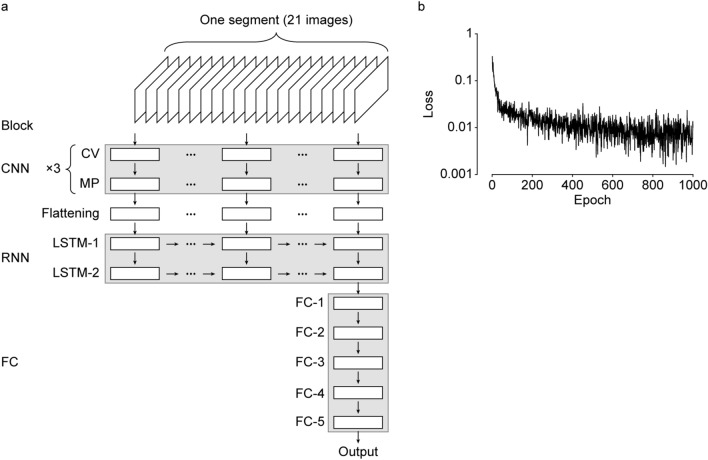
CRNN architecture and training. (**a**) The architecture of CRNN. The images in one segment was separately input into CNN block. The output was flattened and integrated in RNN block and in full connected block. The detailed shapes of output tensor from each layer were shown in Supplementary Table S4. *CV* convolution, *MP* maxpooling, *LSTM* long short-term memory, *FC* fully connected. (**b**) The change of loss value during training.

We constructed a CRNN with three blocks: CNN, RNN, and fully connected (FC) blocks (Fig. 2a). All 21 images in a segment were separately input into the CNN block. Three convolutional layers and a max pooling layer reduced the size of the feature map. The output tensor of the CNN block was flattened and integrated in long short-term memory (LSTM) units in the RNN block. Finally, the FC blocks converged the feature and returned the final output as a decimal value between 0 and 1.

Scratching is a relatively less frequent behaviour compared with others like running, resting, rearing, and grooming. Indeed, scratch frames accounted for less than 2% of the total training dataset. To deal with such imbalanced data, we increased the ratio of the scratching segments in input (called as upsampling) as follows. We randomly selected 1500 segments from the total segments and 100 from the scratching segments in the training dataset. These 1600 segments were flipped and rotated at random for data augmentation and then input to CRNN at once (this input was defined as an epoch). As shown in Fig. 2b, losses for the training dataset continuously decreased over 900 epochs and then reached a plateau. Training was stopped after 1000 epochs.

### Detailed performance evaluation of trained CRNN

**Figure 3:**
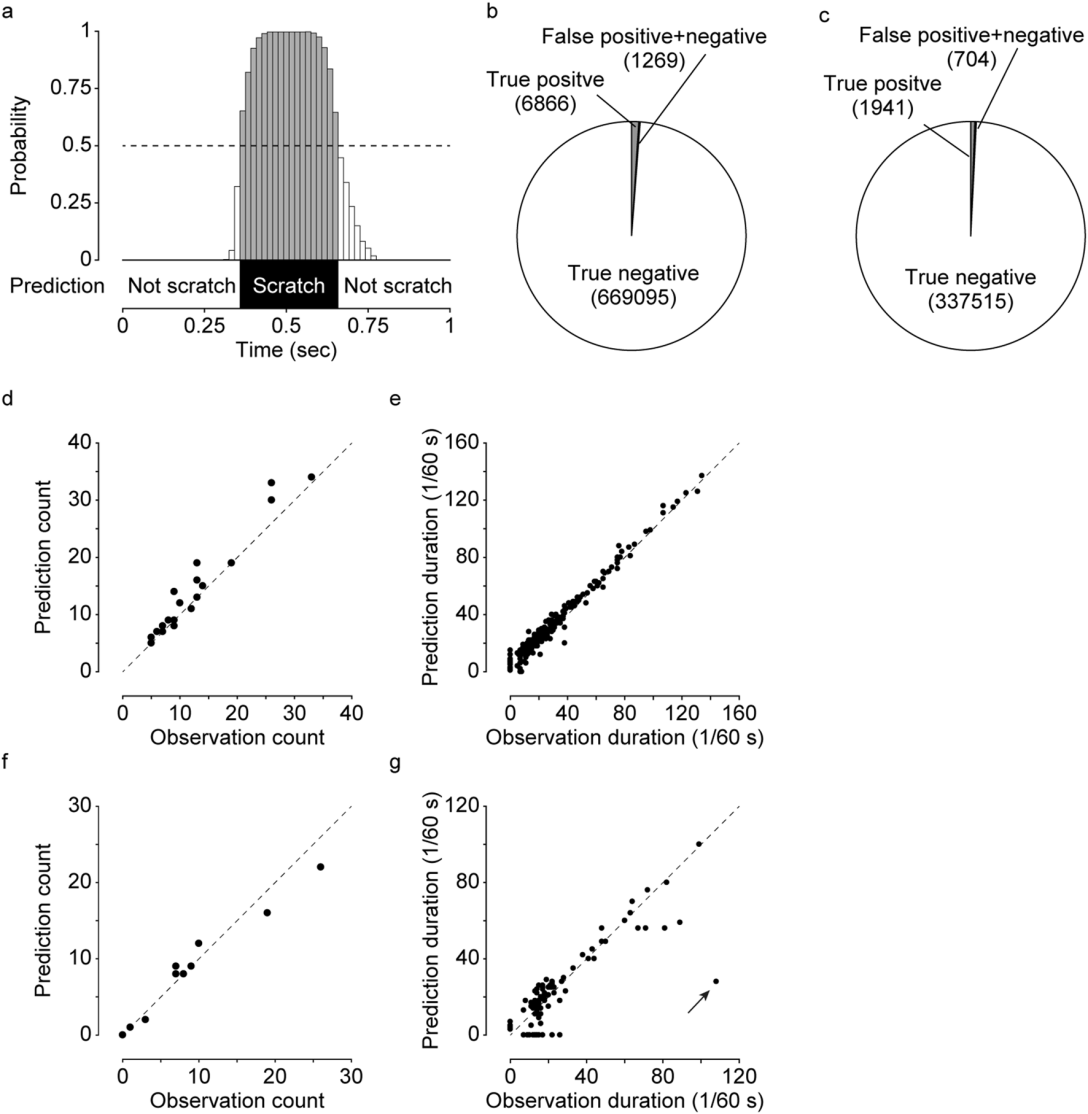
The result of CRNN training. (**a**) The example of CRNN output interpretation. A segment whose CRNN output was more than 0.5 was classified as scratching segment. (**b**) The number of true positive/negative segments and false positive/negative segments in the training dataset. (**c**) The number of true positive/negative segments and false positive/negative segments in the test dataset. (**d**) The comparison of scratching counts in each video file between prediction and observation in the training dataset. (**e**) The comparison of duration time of each scratching event between prediction and observation in the training dataset. (**f**) The comparison of scratching counts in each video file between prediction and observation in the test dataset. (**g**) The comparison of duration time of each scratching event between prediction and observation in the test dataset. Significantly deviated event was indicated as arrow (see Fig. 4d). The dotted lines indicate the line when prediction was equal to observation.

We evaluated the performance of the trained CRNN using the training dataset. For one segment, the CRNN returned a decimal value between 0 and 1, which could be interpreted as the probability to scratch. As shown in Fig. 3a, we classified a segment as scratching when the output value was more than 0.5. For the training dataset, CRNN could correctly predict 97.8% of scratch segments (6866 in 7018) and 99.8% of non-scratch segments (669,095 in 670,212), which corresponded to sensitivity and specificity, respectively (Table 1; Fig. 3b). The positive predictive rate was 86.0% (6866 in 7983), and the negative predictive rate was 99.9% (669,095 in 669,247).

**Table 1:** Confusion matrix for training dataset.

Observation	Prediction		Total
	Scratch	Not scratch	
Scratch	6866	152	7018
Not scratch	1117	669,095	670,212
Total	7983	669,247	677,230

We counted the predicted number of scratching events by trained CRNN in each movie file and compared it with that of the human observation (Obs). We defined the predicted number of scratching events (“Prediction count” in figures) as the number of a series of continuous scratching segment. As shown in Fig. 3d, the predicted number of scratching events was highly correlated with the Obs number of scratching events (r = 0.97). We also calculated the duration for each scratching event and found that the predicted duration correlated with the duration of the Obs (r = 0.99, Fig. 3e). These results clearly indicate that learning was successfully conducted.

We then evaluated the performance of CRNN for the test dataset (Table 2; Fig. 3c). The test dataset was not used for training, and mice in test dataset were also different from those in training dataset (Supplementary Table S1). For the test dataset, CRNN predicted 81.6% of scratch segments (1941 in 2379) and 99.9% of non-scratch segments (337,515 in 337,781). The positive and negative predictive rates were 87.9% (1941 in 2207) and 99.9% (337,515 in 337,953), respectively. The prediction count and duration were also significantly correlated with the Obs count and duration. (r = 0.98 and 0.85 respectively; Fig. 3f,g).

**Table 2:** Confusion matrix for test dataset.

Observation	Prediction		Total
	Scratch	Not scratch	
Scratch	1941	438	2379
Not scratch	266	337,515	337,781
Total	2207	337,953	340,160

### Detailed evaluation of errors

**Figure 4:**
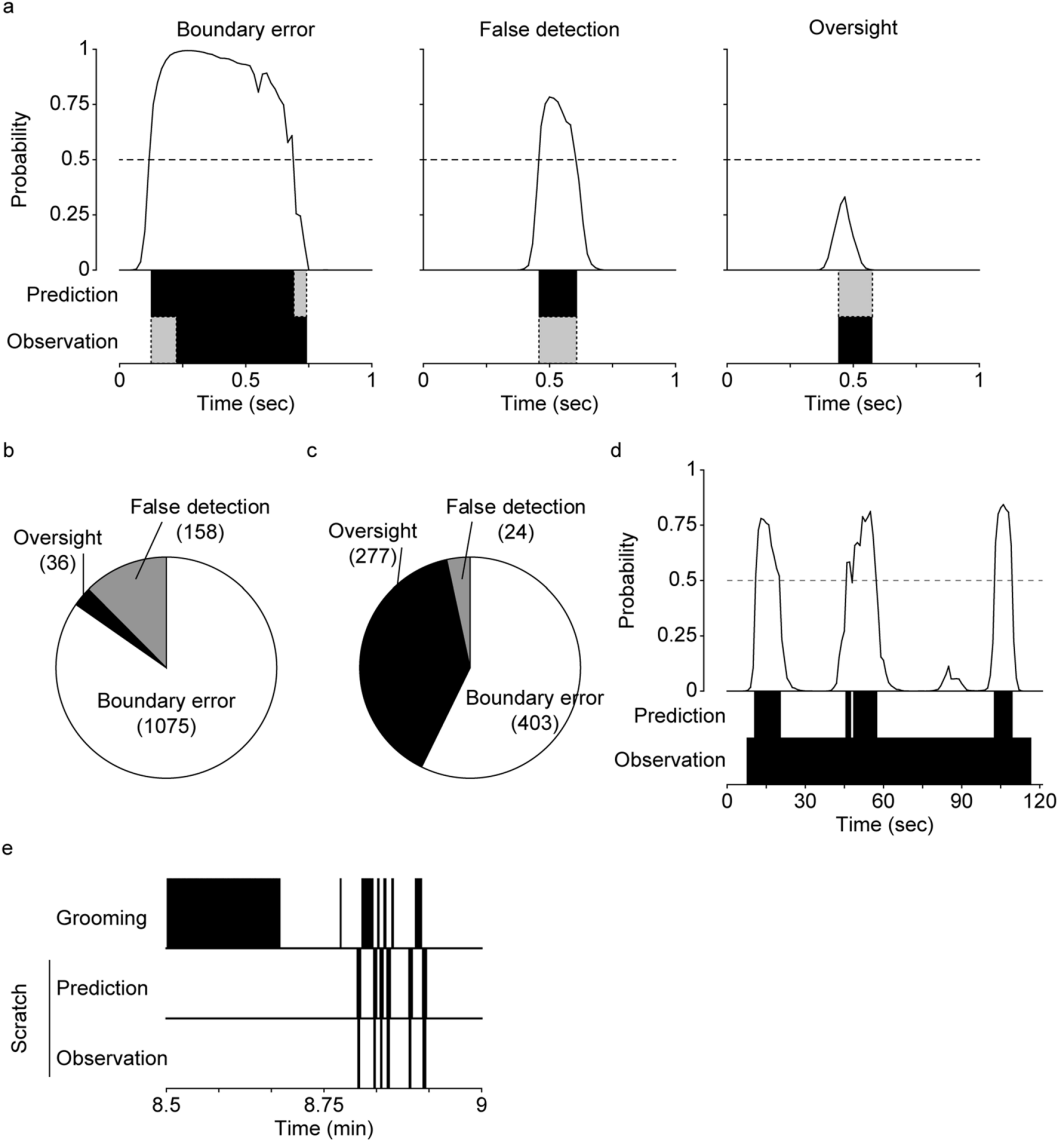
The detailed investigation of error segments. (**a**) Three error types. Black boxes indicate scratching segment in prediction or observation. Grey boxes indicate error segment. (**b**) The number of three errors in the training dataset. (**c**) The number of three errors in the test dataset. (**d**) Detailed investigation of the significantly deviated scratching event (indicated by arrow in Fig. 3g). (**e**) The representative data of grooming and scratching. Black boxes indicate grooming or scratching segment.

We examined the prediction results in detail and classified errors (i.e., false positive and false negative in Fig. 3b,c) into three types: (1) “boundary error,” when the CRNN prediction was generally good, but there was some discrepancy with the Obs regarding when scratching started and/or ended; (2) “false detection,” when the CRNN predicted a scratching event but the mouse did not scratch in reality; (3) “oversight,” when the CRNN predicted no scratching, but the mouse scratched in reality (Fig. 4a). In the training datasets, 85% of the total error segments was boundary error, 12% was false detection, and 3% was oversight (Fig. 4b). In the test datasets, 57% of the total error segments was boundary error, 3% was false detection, and 39% was oversight (Fig. 4c). These results showed that most of false positives and false negatives were boundary errors which were probably caused from the difference in recognition between Obs and CRNN. On the contrary, further improvement may be possible by decreasing the number of false detection and/or oversight segments for CRNN training.

In addition, we analysed a scratching event that showed a large discrepancy between predicted and Obs scratch durations (Fig. 3g, indicated by the arrow). Figure 4d shows detailed predicted probability and Obs data. It clearly shows that while prediction indicated four separate scratching behaviours, the human eye read this as continuous mouse scratching for about 110 s. This may be due to differences in accuracy between human and machine image recognition.

### Differentiation from grooming

Grooming is a common behaviour that mice use to clean their skin and fur. Mice groom themselves by moving their hands along their body, a motion that sometimes resembles scratching. In order to examine whether trained CRNN misclassifies grooming as scratching, we manually labelled grooming events and compared them with prediction using the test dataset (representative example was shown in Fig. 4e). There were 50,147 grooming segments in the test dataset. Among them, only 15 segments were classified as scratching segment by trained CRNN. The CRNN almost perfectly differentiates between scratching and grooming.

### Application to the dinitrofluorobenzene (DNFB)-induced dermatitis model

**Figure 5:**
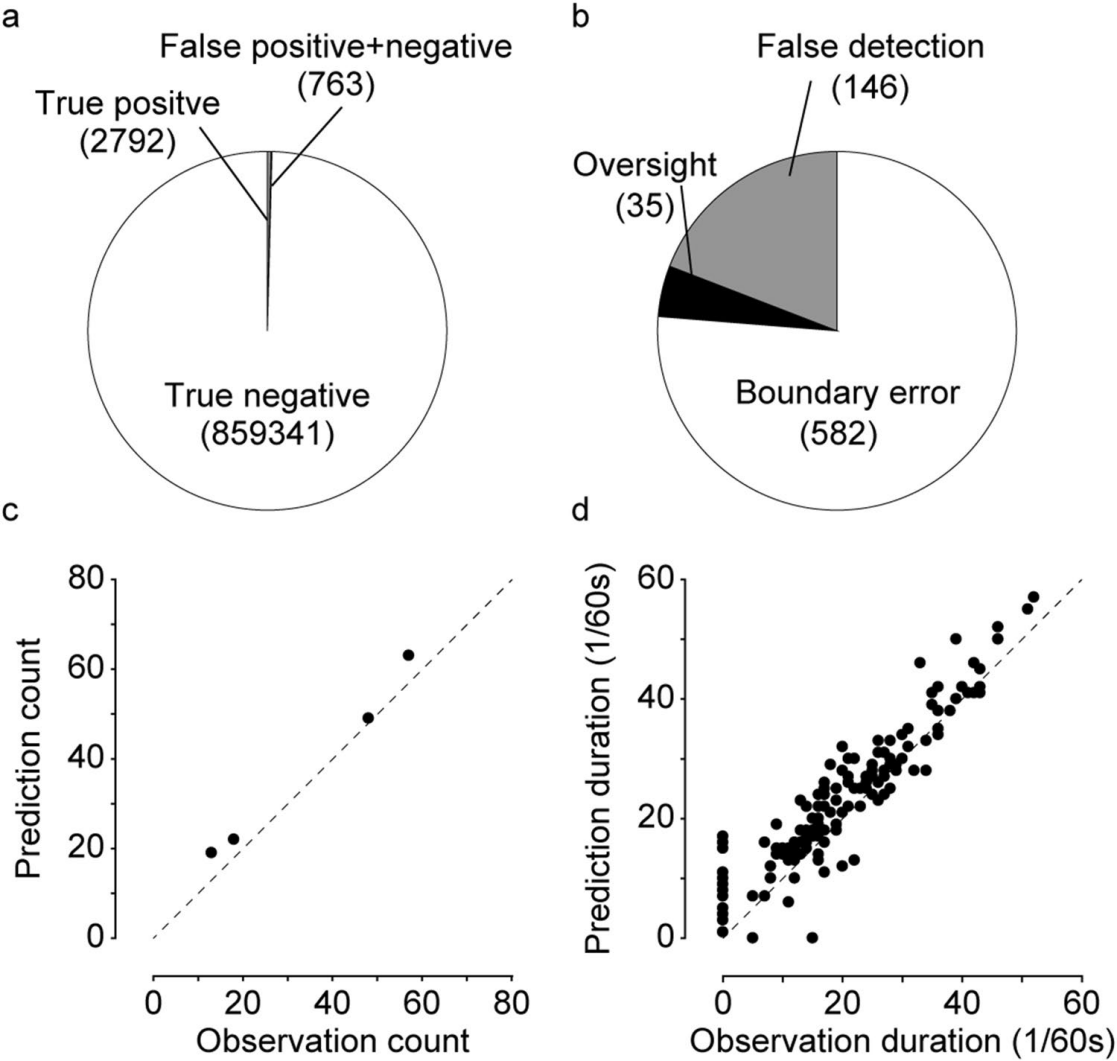
Application to DNFB-induced dermatitis model. (**a**) The number of true positive/negative segments and false positive/negative segments of DNFB-treated mouse video files. (**b**) The number of three errors. (**c**) The comparison of scratching counts in each video file between prediction and observation. (**d**) The comparison of duration time of each scratching event between prediction and observation. The dotted lines indicate the line when prediction was equal to observation.

Finally, we investigated whether our trained CRNN can successfully detect scratching behaviour in a common pathological mouse model. We chose the DNFB-induced dermatitis mouse model; the major manifestation of dermatitis is scratching. BALB/c mice (n = 4, mouse J to M in Supplementary Table S1) were sensitized with DNFB (0.5%/25 μL, applied to ventral skin). Four days later, both ears of the mice were stimulated with DNFB (0.2%/20 μL), and their behaviour was recorded in grey cages for 60 min, beginning immediately after stimulation. As shown in Table 3 and Fig. 5a, CRNN successfully predicted 94.8% of the scratch segments (2792 in 2945) and 99.9% of the non-scratch segments (859,341 in 859,951). We also analysed error type and found that 76.3% of the total error segments was boundary error, 19.1% was false detection, and 4.6% was oversight (Fig. 5b). We finally compared scratching count and duration between prediction and Obs, and, as shown in Fig. 5c,d, there was an excellent correlation between Obs and prediction for scratching count and duration (r = 0.99 and 0.93, respectively). Thus, these results demonstrate that the CRNN can be applied to a common pathological mouse model.

**Table 3:** Confusion matrix for dermatitis-induced scratching.

Observation	Prediction		Total
	Scratch	Not scratch	
Scratch	2792	153	2945
Not scratch	610	859,341	859,951
Total	3402	859,494	862,896

## Discussion

Assessing the behaviour of experimental animals is essential for understanding their physical, mental, and cognitive status. In the present study, we established a novel automated method that can identify scratching in recorded video files using CRNN and further show that it can be used to assess pathological models. It is important to note that our method only requires common equipment, and the program script was written in a freely available programming language.

Many studies have investigated itch sensation in experimental animals. However, they typically use direct observation to detect mouse scratching, which is labour-intensive, and has a low throughput. Automated detection systems are typically more sophisticated but often require specific equipment^4,6^. In the current study, we recorded mouse behaviour using a commercially available handy camera and analysed the images using a common desktop computer with a GPU. In addition, we introduced frame subtraction for image pre-processing. This procedure not only reduces the file size for training and prediction but also cancels out any background noise. We utilized different type of cages for recording the LPA-treated mice (black arena) and that for DNFB-treated mice (grey cage). The trained CRNN could predicted mouse scratching behavior induced by DNFB correctly. Although further verification is required, this technology has some versatility.

CNN is known for its significant performance in image recognition tasks. In the field of biology, several studies have highlighted the usefulness of CNN-based algorithms. For example, Pereira et al. showed that CNN could estimate the position of body parts from a single image^9^. Another study enabled pose estimation with very little training data by combining CNN and transfer learning^10^. Schofield et al. also successfully identified the faces of chimpanzees using CNN^14^. We also attempted to establish CNN or shallow CRNN with less layers to predict mouse scratching behaviour. However, they represented limited performance as shown in Supplementary Note and Supplementary Fig. S1. Since animal behaviour is composed of sequential motions, we assumed that the combination of CNN and multiple RNN layers can be better strategy for behavioural detection.

We trained the CRNN using sequential pictures obtained from each movie in which mice were administrated LPA to the skin on their back. The trained CRNN successfully predicted LPA-induced scratching. Notably, it could also classify DNFB-induced scratching when DNFB was topically applied to mouse ears. These results suggest that the behaviour of scratching the back and ears was sufficiently similar for CRNN. However, it is still unclear whether this CRNN can identify other types of scratching, including systemic scratching induced by cholestasis or nose/mouth scratching with the forelimbs, such as that caused by food allergies^15,16^.

There are some limitations in the system developed in this study. In the preliminary analysis, we determined important parameters to establish better neural network as shown in Supplementary Note; binarization threshold, the number of frames per segment, the upsampling ratio, and NN architecture. In addition to these tunings, image acquisition using a high-speed camera and selection of training dataset can improve the accuracy. Researchers often utilized black mice including C57BL/6 as well as white mice. We found that current CRNN trained with white BALB/c mice dataset represented relatively lower performance in the detection of LPA-induced scratching in black C57BL/6 mice (sensitivity: 22.1%, Supplementary Fig. S2; Supplementary Table S5). Training dataset obtained from various mouse strains may expand the abilities of this method.

We specifically focused on scratching among the various animal behaviours. However, we predict that the procedure discussed here could be applied to other behaviours, such as grooming, rearing, resting, and feeding. In addition, the method could be applied to long-term studies (e.g., 24 h). As a result, it is possible that this automated technique could be used to detect and classify any and all mouse behaviours during a single day, which would provide novel insights into animal ethology.

In conclusion, we have established a novel and accurate scratching detection method using CRNN, which can also be used to assess pathological models.

## Methods

### Mice

BALB/c mice (12–16 weeks old; male and female) and C57BL/6J mice (8–10 weeks old; male and female) were purchased from Charles River Japan (Yokohama, Japan). All experiments were approved by the institutional Animal Care and Use Committee at the University of Tokyo (P18-067 and P19-079). Animal care and treatments were performed in accordance with the guidelines outlined within the Guide to Animal Use and Care of the University of Tokyo.

### Scratch induction by lysophosphatidic acid (LPA) treatment

A pruritogen, LPA (200 nmole/site/25 μL; Avanti Polar Lipids, Alabaster, AL, US), was intradermally injected to the back of BALB/c mice (2 site/mouse, n = 9)^17,18^. Immediately after injection, mice were placed into a black square arena (40 cm × 40 cm × 27 cm), and their behaviours were recorded for 30–60 min using a handy camera (HDR-CX720V, Sony, Tokyo, Japan) set at a height of 150 cm above the arena. Detailed recording conditions were as follows: frame rate, 60 Hz; resolution, 1920 × 1080 pixel, 24-bit colour. We used thirty of nine-minute video files in which mice scratched several times. These video files were split into training (20 files, mouse A to F in Supplementary Table S1) and test (10 files, mouse G to I in Supplementary Table S1) datasets. We note that mice in training dataset were different from those in test dataset. The training dataset was used for CRNN training and the test dataset for performance evaluation.

### Dermatitis model

2,4-Dinitrofluorobenzene (DNFB, 0.5% in 25 μL; NACALAI TESQUE, INC., Kyoto, Japan) was applied to the shaved ventral skin of mice (n = 4, mouse J to M in Supplementary Table S1). Four days later, DNFB (0.2% in 20 μL) was applied to both ears. Behaviour was recorded for 60 min in grey rectangular parallelepiped cages (37 cm × 25 cm × 22 cm), beginning immediately after DNFB application.

### Image pre-processing

Images of all frames of each video file were obtained. The absolute difference of each pixel between two adjoining frames was calculated (Fig. 1a). Differential images were cropped into a square shape (300 × 300 pixel) around the geometric centre of the mouse, which was independently calculated with previously described method^13^. The images were grey-scaled and binarized using a pre-defined threshold. In the preliminary analysis, we tried three binarization threshold values (15, 25, and 35) among 0–255 and finally set 25 as threshold which showed best performance. These procedures were applied to all frames in each video except the first one.

### Manual behaviour detection

Scratching behaviour was defined as the rapid, repetitive, and back-and-forth movement of the hindlimb toward the injection site. We played the recorded video file in slow motion and identified scratching behaviour. We then scrutinized each frame and determined when it started and ended. All frames in all video files are labelled and checked by two researchers independently. These labels were named as human observation (Obs). For convenience, frames where the mouse was scratching were labelled as 1 and other frames were labelled as 0. Grooming was also manually identified in the test dataset video files.

### Architecture of CRNN

The architecture of CRNN (described as architecture C in Supplementary Note) consists of three main parts: CNN, RNN, and FC blocks (Fig. 2a; Supplementary Table S4). The CNN block is composed of three alternately arranged convolutional layers (CV-1–CV-3; 32 filters, 3 × 3 kernel size, 1 × 1 stride, ReLU activation) and max pooling layers (MP-1–MP-3; 2 × 2 pooling size, 2 × 2 stride). The RNN block is composed of two LSTM layers (LSTM-1 and LSTM-2; 256 units, ReLU activation). The FC block is composed of five FC layers (FC-1–FC-5; 128, 128, 32, 8, and 1 unit in sequence). ReLU activation was used in the FC-1–FC-4 layers and sigmoid activation was used in the FC-5 layer. Twenty percent of the units in the FC-1 and FC-2 layers were randomly dropped during training. The shape of the output tensor from each layer is given in Supplementary Table S4. The other NN architectures in Supplementary Note (architecture A and B) were constructed by removing several layers from architecture C (Supplementary Tables S2, S3).

### CRNN training

For a frame at time *t*, 21 pre-processed images were collected from *t − 10* to *t* + *10*, which were named the “segment” at time *t*. Segments were labelled with the value of the frame label at time *t*. Therefore, one segment had 21 serial images (300 × 300 pixel) and one label (0: not scratch, 1: scratch). For training, 20 video files were used (training dataset). Fifteen hundred segments were randomly selected from the total segments, and 100 segments were selected from the scratch segments per epoch, allowing for duplicates. This upsampling ratio (1500/100) was determined by preliminary analyses where we tried 1600/0, 1500/100, and 1400/200 and found 1500/100 was the best. Images within these 1600 segments were randomly flipped and rotated for data augmentation; then, they were resized to 200 × 200 pixel and input into CRNN. An ADAM optimizer with a 10^–4^ learning rate and binary cross entropy loss function were used.

### Scratching prediction

For prediction, each segment was input into the trained CRNN without data augmentation. CRNN outputs a decimal value between 0 and 1 for each segment, which is interpreted as the possibility to scratch. We classified a segment as scratching when its output value was more than 0.5.

### Computer hardware

The calculations described above were conducted on a desktop computer equipped with an Intel Core i7-8086K CPU, 32 GM RAM, and NVIDIA GeForce GTX 1080 Ti (11 GB) GPU. Image pre-processing was conducted using an image processing library (OpenCV, version 3.4.7) in Python. CRNN training and prediction were conducted using Keras library (version 2.2.4) in Python.

## Supplementary Information

Supplementary Information

## Data Availability

The datasets generated during the current study are available from the corresponding author on reasonable request.
