# Supplementary material for: Automated detection of mouse scratching behaviour using convolutional recurrent neural network
Source: Sci Rep. 2021 Jan 12;11:658. doi: 10.1038/s41598-020-79965-w (PMC7803777; doi:10.1038/s41598-020-79965-w)
Supplement: Supplementary file 1 — Supplementary Information [file 41598_2020_79965_MOESM1_ESM.docx]

**Title:**

**Automated detection of mouse scratching behaviour using convolutional recurrent neural network**

**Authors’ name and affiliation:**

Koji Kobayashi^1^, Seiji Matsushita^1^, Naoyuki Shimizu^1^, Sakura Masuko^1^, Masahito Yamamoto^2^, and Takahisa Murata^1*^.

1. Department of Animal Radiology, Graduate School of Agricultural and Life Sciences, The University of Tokyo, Tokyo, Japan.

2. Autonomous Systems Engineering Laboratory, Graduate School of Information Science and Technology, Hokkaido University, Sapporo, Japan

**Corresponding author’s information:**

*Corresponding author: Takahisa Murata, D.V.M., Ph.D.

Department of Animal Radiology, Graduate school of Agricultural and Life Sciences, The University of Tokyo

Address: 1-1-1, Yayoi, Bunkyo-ku, Tokyo 113-8657, Japan.

E-mail: amurata@mail.ecc.u-tokyo.ac.jp

Telephone: +81-3-5841-7247, Fax: +81-3-5841-8183.

**Supplementary Note**

**Optimization of NN architecture**

In order to select the NN architecture, we constructed several NNs from simple CNN to deep CRNN as follows, A: CNN only (Supplementary Fig. S1a); B: CRNN with fewer layers (Supplementary Fig. S1b); C: deep CRNN (Fig. 2a). The detailed information about these architectures is given in Supplementary Table S2-S4. Three NNs (A-C) were trained with training dataset and their performance were evaluated using same dataset. Among three types of NNs, architecture C exhibited best performance which showed least false positive/negative segments (Fig. 3a, Supplementary Fig. S1d, and S1e). Therefore, we employed this deep CRNN architecture in this manuscript.

**Optimization of the number of images per segment**

We also tried to change the number of images per segment. When we grouped 11 images per segment (Supplementary Fig. S1c), the accuracy of prediction was greatly decreased (Supplementary Fig. S1f and Fig. 2a). We also tried 41 frames per segment, but the CRNN training with them could not be executed since it exceeded the GPU memory capacity of our PC. Based on these results, we set the number of frames per segment as 21 in this manuscript.

**Supplementary figures**

**
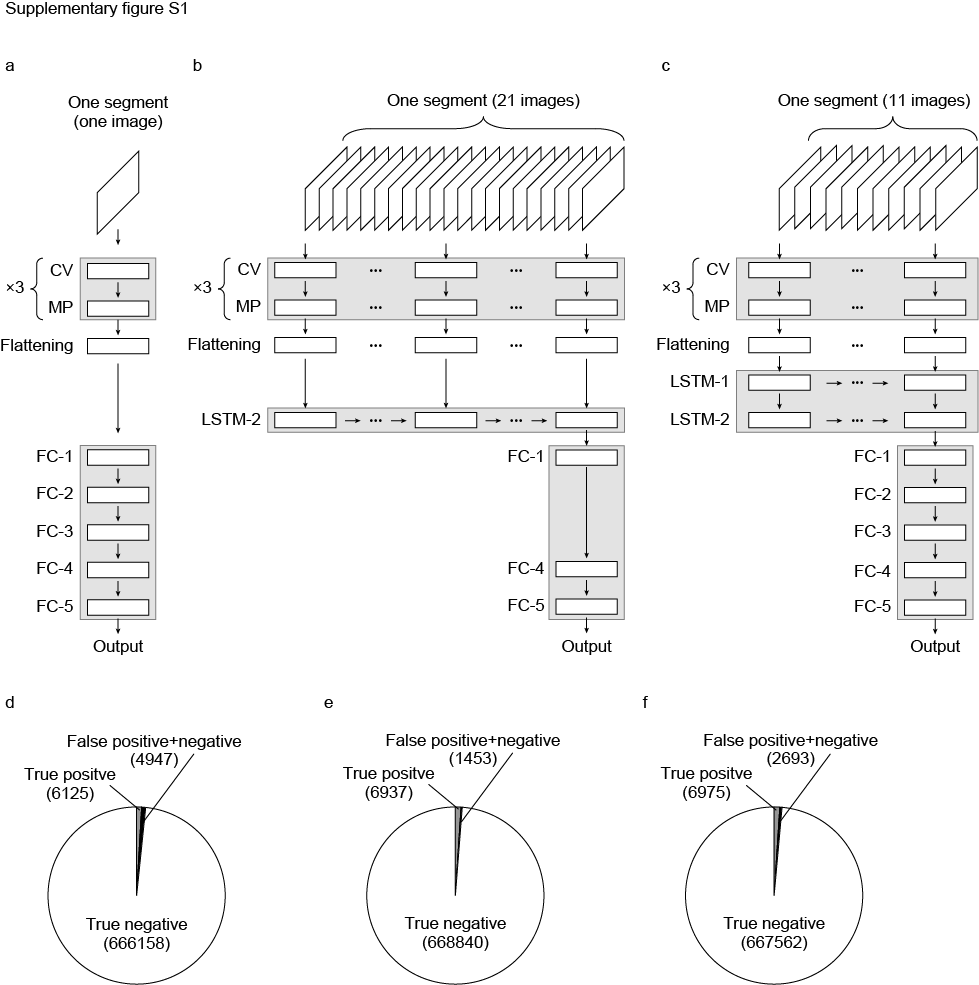
**

**
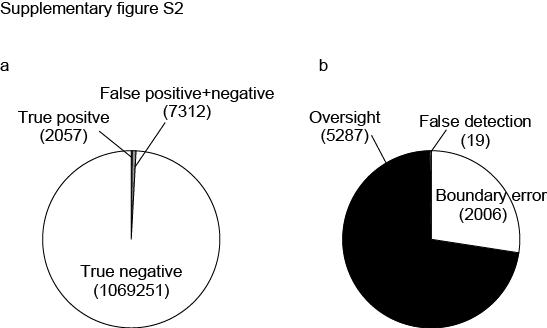
**

**Supplementary figure legends**

**Supplementary Fig. S1. The optimization of NN architecture.**

(a) The architecture of CNN only NN (architecture A). (b) The architecture of shallow CRNN (architecture B). (c) The architecture of deep CRNN with 11 images per segment. (d-f) The number of true positive/negative segments and false positive/negative segments of architecture A (d), architecture B (e), and architecture C with 11 images per segment (f).

**Supplementary Fig. S2. Application to C57BL/6J mice.**

LPA (200 nmole/site/25 μL) was intradermally injected to the back of C57BL/6J mice (2 site/mouse, n = 5). Immediately after the injection, mice were placed into a white cage (37 cm × 25 cm × 22 cm) and their behaviours were recorded for 60 minutes as described in Methods.

(a) The number of true positive/negative segments and false positive/negative segments in the LPA-treated C57BL/6J mice dataset. (b) The number of boundary errors, false detection, and oversight segments.

**Supplementary tables**

Supplementary Table S1. Datasets information

| Dataset | Video No. | Mouse No. | Time (min:sec) | Scratch count |
| --- | --- | --- | --- | --- |
| LPA training | 01 | A | 9:49 | 26 |
|  | 02 | A | 9:23 | 26 |
|  | 03 | A | 9:09 | 13 |
|  | 04 | B | 9:24 | 6 |
|  | 05 | B | 9:18 | 7 |
|  | 06 | B | 9:23 | 14 |
|  | 07 | C | 9:27 | 5 |
|  | 08 | C | 9:26 | 9 |
|  | 09 | C | 9:15 | 7 |
|  | 10 | D | 10:21 | 9 |
|  | 11 | D | 9:27 | 19 |
|  | 12 | E | 9:15 | 5 |
|  | 13 | E | 9:29 | 12 |
|  | 14 | E | 9:16 | 9 |
|  | 15 | E | 9:19 | 13 |
|  | 16 | F | 9:17 | 13 |
|  | 17 | F | 9:45 | 5 |
|  | 18 | F | 9:25 | 10 |
|  | 19 | F | 9:21 | 33 |
|  | 20 | F | 9:21 | 8 |
| LPA test | 21 | G | 9:26 | 10 |
|  | 22 | G | 9:42 | 19 |
|  | 23 | G | 9:27 | 9 |
|  | 24 | G | 9:27 | 7 |
|  | 25 | H | 9:21 | 0 |
|  | 26 | H | 10:01 | 7 |
|  | 27 | H | 9:23 | 26 |
|  | 28 | I | 9:23 | 8 |
|  | 29 | I | 9:20 | 1 |
|  | 30 | I | 9:21 | 3 |
| DNFB | 31 | J | 60:00 | 58 |
|  | 32 | K | 60:00 | 13 |
|  | 33 | L | 60:00 | 48 |
|  | 34 | M | 60:00 | 18 |

Supplementary Table S2. The architecture of neural network A

| Block | Layer | Shape of output tensor |
| --- | --- | --- |
| Convolution | CV-1 | 21×200×200×32 |
|  | MP-1 | 21×100×100×32 |
|  | CV-2 | 21×100×100×32 |
|  | MP-2 | 21×50×50×32 |
|  | CV-3 | 21×50×50×32 |
|  | MP-3 | 21×25×25×32 |
| Tensor flattening | Flatten | 21×20000 |
| FC | FC-1 | 128 |
|  | FC-2 | 128 |
|  | FC-3 | 32 |
|  | FC-4 | 8 |
|  | FC-5 | 1 |

Supplementary Table S3. The architecture of neural network B

| Block | Layer | Shape of output tensor |
| --- | --- | --- |
| Convolution | CV-1 | 21×200×200×32 |
|  | MP-1 | 21×100×100×32 |
|  | CV-2 | 21×100×100×32 |
|  | MP-2 | 21×50×50×32 |
|  | CV-3 | 21×50×50×32 |
|  | MP-3 | 21×25×25×32 |
| Tensor flattening | Flatten | 21×20000 |
| RNN | LSTM-1 | 256 |
| FC | FC-1 | 128 |
|  | FC-2 | 8 |
|  | FC-3 | 1 |

Supplementary Table S4. The architecture of neural network C

| Block | Layer | Shape of output tensor |
| --- | --- | --- |
| Convolution | CV-1 | 21×200×200×32 |
|  | MP-1 | 21×100×100×32 |
|  | CV-2 | 21×100×100×32 |
|  | MP-2 | 21×50×50×32 |
|  | CV-3 | 21×50×50×32 |
|  | MP-3 | 21×25×25×32 |
| Tensor flattening | Flatten | 21×20000 |
| RNN | LSTM-1 | 21×256 |
|  | LSTM-2 | 256 |
| FC | FC-1 | 128 |
|  | FC-2 | 128 |
|  | FC-3 | 32 |
|  | FC-4 | 8 |
|  | FC-5 | 1 |

Supplementary Table S5. Confusion matrix for C57BL/6 mice dataset

|  | | Prediction | | Total |
| --- | --- | --- | --- | --- |
|  |  | Scratch | Not scratch |  |
| Observation | Scratch | 2057 | 7230 | 9287 |
|  | Not scratch | 82 | 1069251 | 1069333 |
| Total | | 2139 | 1076481 | 1078620 |
